# Giant Condylomata Acuminata of Buschke-Lowenstein Associated With Paraneoplastic Hypercalcemia

**DOI:** 10.1177/2324709618758348

**Published:** 2018-02-15

**Authors:** Fredrick Venter, Arash Heidari, Macsen Viehweg, Mark Rivera, Piruthiviraj Natarajan, Everardo Cobos

**Affiliations:** 1 Kern Medical Center, Bakersfield, CA, USA

**Keywords:** giant condylomata acuminata, Buschke Lowenstein tumor, squamous cell carcinoma, paraneoplastic hypercalcemia, genital warts, HPV vaccination, E6 and E7 oncogenes, human papillomavirus

## Abstract

Low-risk human papillomavirus types 6 and 11 can manifest as giant condylomata acuminata (GCA) of Buschke-Lowenstein. Up to 50% of GCA can slowly progress over years to fungating, invasive tumors. The malignant potential is attributed to unique immune evading abilities of the human papillomavirus. A 42-year-old male presented with pain and foul-smelling discharge from his genital warts. The histopathological examination of the mass showed invasive squamous cell carcinoma, and it was associated with paraneoplastic hypercalcemia. The timely removal of long-standing GCA in order to prevent a carcinomatous transition is a priority.

## Introduction

A genital wart is a painless growth over the skin surface frequently associated with human papillomavirus (HPV) infection 6 and 11.^1^ More than 80% of genital warts are associated with low-risk HPV subtypes 6 and 11 and the rest with high-risk HPV subtypes 16, 18, 52, and 56.^2^ Multiple high- and low-risk viral subtype coinfection can occur at the same time.^3^ Even though most of the warts are considered benign, a long-standing genital wart can turn malignant due to the dynamics between the virus and the immunologic response of the host.^4^ More than 90% of the genital warts regress within 18 months whereas others persist and recur over time. Immune deficiency status augments the malignant potential of the giant warts.^5^ The size of development of genital warts vary among individuals, and the one that grows to a considerable size is called giant condylomata acuminata (GCA). GCA has a 0.1% incidence rate in general population, with a 50% recurrence rate. Predominantly men, those uncircumcised, smokers, and those who had sexual intercourse before 16 years of age are at increased risk.

## Case Presentation

A 42-year-old Native American male was admitted at the emergency department with nausea, vomiting, abdominal pain localized to groin, and associated with foul-smelling discharge. The genital warts diagnosed at the age of 17, deferring excision, had reached a considerable size over the years. For the past 5 months, the friable mass rapidly increased in size and expanded its ulcerated borders and was associated with occasional serous/bloody discharge. He had unintended weight loss of 100 pounds in the past year, and in the past few months, he suffered fatigue and loss of appetite.

**Figure 1.**
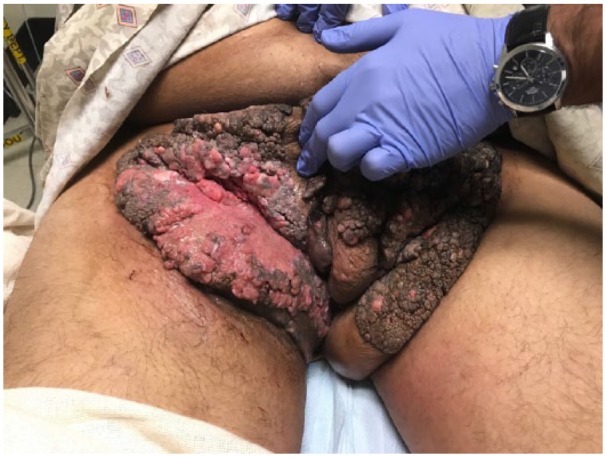
Condyloma acuminata of the groin and pelvic region disfiguring the male genitalia.

A large irregular mass over bilateral inguinal regions involved the penis, scrotum, and perineum measuring to 31 × 17 × 6 cm by its longest dimensions. Right inguinal ulcer over the mass measured to 11 × 7 × 5 cm. The erythematous ulcer base had multiple fissures draining foul-smelling purulent discharge (Figure 1).

**Figure 2.**
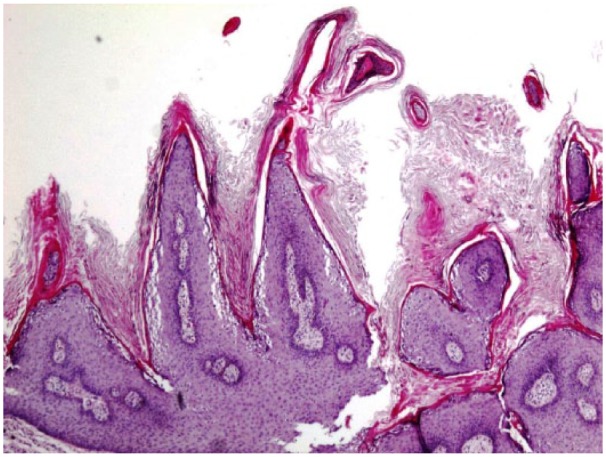
Hematoxylin and eosin 4× stain of biopsy showing papillomatosis and hyperkeratosis, defining condyloma acuminata.

**Figure 3.**
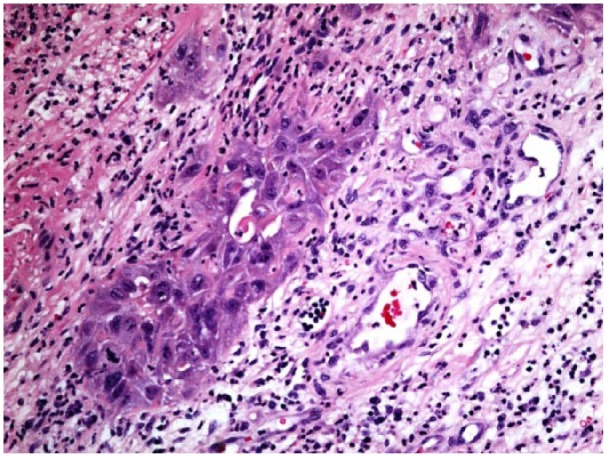
Hematoxylin and eosin 20× stain of condyloma acuminatum showing mitotic figures and squamous cell carcinoma invasion.

The serum blood calcium level at admission was 13.8 mg/dL (normal = 8.5-10.2 mg/dL) with PTHrP 24.1 pmol/L (normal = <2.0 pmol/L) suggesting secondary hyperparathyroidism. Herpes simplex virus (HSV)-1 results are positive with IgG > 5 OD ratio. HTLV and HIV subtypes were negative. Biopsy hematoxylin and eosin stain showed infiltrating hyperchromatic squamous cells, enlarged nuclei, and higher nuclear/cytoplasmic ratio; hyperkeratotic spears and acanthosis were also present (Figures 2 and 3). On computed tomography scan of pelvis, marked skin thickening and irregularity in the perineal/inguinal/upper thigh regions and the right inguinal region was associated with inflammation and fistulous tracts. Computed tomography scan of chest was negative for metastasis. Paraneoplastic moderate hypercalcemia was managed by zoledronic acid intravenous infusion. Calcium level was corrected to 8.3 mg/dL at discharge. Surgeons decided against resection of the mass considering the size and site of the lesion. The oncologist team started on pembrolizumab for the inoperable mass.

## Discussion

Sexually transmitted HPV invades the genital epidermal basal layer cells through micro-abrasions. HPV completes its life cycle outside the genital epithelial basement membrane, and the E7 gene of HPV impairs the antigen presenting cells in the skin, enabling the virus to stay undetected for extended periods of time. Low viral protein expression and impaired viral epitope presentation to cytotoxic T lymphocytes evades the host immune response.^6^ The E6 and E7 oncogenes impair the host cells to induce telomerase resulting in cellular immortalization of the infected cells.^7^ The E6 and E7 oncogenes also induce chronic oxidative stress within the HPV-infected cells, increasing the susceptibility to DNA damage and paving the way for carcinogenesis.^8^ It has been found that coinfection with HIV/HSV-1 enhances the oncogenesis of HPV, and our patient being positive for HSV-1 IgG is susceptible for this carcinomatous transition.^9,10^ Squamous cell clusters with high mitotic activity and keratinization among the condyloma stroma in histopathological examination suggest nests of carcinomatous transformation. An association of carcinoma with hypercalcemia usually indicates a poor prognosis. The moderate hypercalcemia in our patient was attributed to PTHrP secreted by tumor cells. The PTHrP resembles parathyroid hormone as they both have the common first 13 N-terminal amino acids, and PTHrP binds to parathyroid hormone receptors adversely activating various organs with most of the damage to the bones with its osteolytic action resulting in hypercalcemia.^11^ Squamous cell carcinoma of the anogenital area is usually a slow growing tumor, but the rate of tumor progression in our patient within the past few months could suggest an aggressive phenotypic change. Even though the squamous cell carcinoma can metastasize locally, metastatic spread was negative until the last evaluation. The current evidence suggests no easier and reliable way of screening the warts with carcinogenic potential at an early stage, but an early removal of the wart could potentially avoid the GCA. A pre-exposure HPV vaccination could reduce the incidence in susceptible high-risk populations, and a post-exposure vaccination can potentially prevent wart/condylomata formation.^12^ Vaccines augment the production of CD8+T cells, synthesizing perforin, can kill infected basal cells, and regress the precancerous lesions.^13^

## Conclusion

Squamous cell malignant transformation of anogenital warts is rare, and an associated paraneoplastic hypercalcemia is first to be reported. Coinfection with other viruses enhance the oncogenic ability of HPV. Since the oncogenic process is slow, an early removal of the warts when noticed by the patient could be useful to prevent a GCA. Pre-exposure and post-exposure vaccines play a role in preventing HPV warts and reducing the viral load in the infected.
